# Issue Information

**DOI:** 10.1002/phy2.114

**Published:** 2013-09-23

## Aims and Scope

**Physiological Reports** is an online only, open access journal that will publish peer-reviewed research across all areas of basic, translational and clinical physiology and allied disciplines. *Physiological Reports* is a collaboration between The Physiological Society and the American Physiological Society, and is therefore in a unique position to serve the international physiology community through quick time to publication while upholding a quality standard of sound research that constitutes a useful contribution to the fi eld. Papers will be accepted solely on the basis of scientifi c rigor, adherence to technical and ethical standards, and evidence that the study is suffi ciently well-conceived and the data support the conclusions.

*Physiological Reports* is interested in cellular and molecular studies as well as intact tissues, model organisms and translational studies. We welcome the work of biomedical scientists, whose studies incorporate physiological fi ndings. Papers with negative data or confi rmatory results are acceptable as long as they are well conceived. Papers concerned with modeling or new methods are acceptable as long as they are of physiological interest. *Physiological Reports* does not publish case reports or clinical trials or unsolicited review articles. For further information, see the journal's full scope statement available at http://onlinelibrary.wiley.com/journal/10.1002/(ISSN)2051-817X/homepage/ProductInformation.html

## Open Access and Copyright

All articles published by *Physiological Reports* are fully open access: immediately freely available to read, download and share. All *Physiological Reports* articles are published under the terms of the Creative Commons Attribution (CC BY) License, which permits use, distribution and reproduction in any medium, provided the original work is properly cited.

Copyright on any research article in a journal published by *Physiological Reports* is retained by the author(s). Authors grant Wiley a license to publish the article and identify itself as the original publisher. Authors also grant any third party the right to use the article freely as long as its integrity is maintained and its original authors, citation details and publisher are identifi ed. Further information about the open access license and copyright can be found at http://www.wileyopenaccess.com/details/content/12f25db4c87/Copyright–License.html.

## Purchasing Print Reprints

As this is an open access journal, you have free, unlimited access to your article online. However, if you wish to obtain printed reprints, these may be ordered online: https://caesar.sheridan.com/reprints/redir.php?pub=10089&acro=PHY2

## Disclaimer

The Publisher and Editors cannot be held responsible for errors or any consequences arising from the use of information contained in this journal; the views and opinions expressed do not necessarily refl ect those of the Publisher and Editors, neither does the publication of advertisements constitute any endorsements by the Publisher and Editors of the products advertised.

Wiley Open Access articles posted to repositories or websites are without warranty from Wiley of any kind, either express or implied, including, but not limited to, warranties of merchantability, fi tness for a particular purpose, or non-infringement. To the fullest extent permitted by law Wiley disclaims all liability for any loss or damage arising out of, or in connection with, the use of or inability to use the content.

**Editor-in-Chief**

Susan Wray, Ph.D.

*University of Liverpool, UK*.

**Deputy Editor-in-Chief**

Thomas Kleyman, M.D.

University of Pittsburgh, USA

Address correspondence to the Editorial Office: physiologicalreports@wiley.com

**Associate Editors**

Julian Davis

University of Manchester, United Kingdom

Gareth Leng

University of Edinburgh, United Kingdom

Meena Rao

University of Illinois at Chicago, USA

Larissa Shimoda

Johns Hopkins School of Medicine, USA

**Consulting Editors**

Kim E Barrett

University of California San Diego, USA

John R G Challis

University of Toronto, Canada

Mark T Nelcon

University of Vermont, USA

Scott M O'Grady

University of Minnesota, USA

Quentin J Pittman

University of Calgary, Canada

John R Solaro

University of Illinois at Chicago, USA

**Editorial Board Members**

Roger Adan

University Medical Center Utrecht, Netherlands

Waddah A Alrefai

University of Illinois, USA

Nadia Ameen

Yale University, USA

Tracy Baker-Herman

University of Wisconsin-Madison, USA

Jerome Bertherat

Hôpital Cochin, France

Andrew M Blanks

Warwick Medical School, United Kingdom

Mitsi A Blount

Emory University, USA

Theirry Capiod

Université Paris Descartes, France

Jorge A Carvajal

Pontifical University of Chile, Chile

Eugene B Chang

University of Chicago, USA

Chen Chen

University of Queensland, Australia

Mary E Choi

Harvard Medical School, USA

Alan Chun-Yao Pao

Stanford University, USA

Tony Coll

Addenbrooke's Hospital, United Kingdom

Hugo de Jonge

Erasmuc MC, Netherlands

Katharine Dibb

University of Manchester, United Kingdom

Caroline Dart

University of Liverpool, United Kingdom

Primal De Lanerolle

University of Illinois, USA

Suzanne L Dickson

University of Gothenburg, Sweden

Paulina Donoso

Universidad de Chile, Chile

Pradeep K Dudeja

University of Illinois, USA

Dominique Eladari

Inserm Institut, France

Robert A Fenton

Aarhus University, Denmark

Candice D Fike

Vanderbilt University Medical Center, USA

Peter W Flatman

University of Edinburgh, United Kingdom

Alison J Forehead

University of Cambridge, United Kingdom

Rob Fowkes

The Royal Veterinary College, United Kingdom

Jørgen Frøkiær

Aarhus University, Denmark

Catherine M Fuller

University of Alabama, USA

John Funder

Prince Henry's Institute, Australia

Jeffrey Garvin

Case Western Reserve University, USA

Sonia Gasparini

Louisiana State University Health Science Center, USA

Ravinder Gill

University of Illinois at Chicago, USA

Robb Glenny

University of Washington, USA

Dave Grattan

University of Otago, New Zealand

Lucy Green

Southampton General Hospital, United Kingdom

Valery Grinevich

University of Heidelberg, Germany

Susan J Gunst

Indiana University, USA

Dan Halm

Wright State University, USA

Kirk L Hamilton

University of Otago, CA

Jules Hancox

University of Bristol, United Kingdom

Lisa M Harrison-Bernard

Louisiana State University Health Sciences Center, USA

Chris Howarth

United Arab Emirates University, United Arab Emirates

Kenichiro Kitamura

Kumamoto University, Japan

Nateetip Krishnamra

Mahidol University, Thailand

Corne J Kros

University of Sussex, United Kingdom

Wolfgang M Kuebler

University of Toronto, Canada

Tim Lahm

Indiana University, USA

Kathryn Lampin

University of Iowa, USA

Florian Lang

University of Tuebingen, Germany

Rachel Lennon

University of Manchester, United Kingdom

Mike Ludwig

University of Edinburgh, United Kingdom

He-Ping Ma

Emory University School of Medicine, USA

Heimo Mairbaurl

University Hospital Heidelberg, Germany

John McCarron

University of Strathclyde, United Kingdom

Fiona McDonald

University of Otago, New Zealand

Alicia A McDonough

Keck School of Medicine, USA

Onno Meijer

Leiden University Medical Centre, Netherlands

Marilyn P Merker

Medical College of Wisconsin, USA

Rory Morty

University Medical Center of Giessen, Germany

John Mullins

University of Edinburgh, United Kingdom

Kazuhiro Nakamura

Kyoto University, Japan

Anjaparabanda P Naren

University of Tennessee, USA

Leif D Nelin

The Research Institute at Nationwide Children's Hospital, USA

Deborah J Nelson

University of Chicago, USA

Ernst Niggli

University of Bern, Switzerland

Paul O'Connor

Georgia Regents University, USA

Scott M O'Grady

University of Minnesota, USA

David B Pearse

Johns Hopkins Medicine, USA

Pontus B Persson

Universitätsmedizin Berlin, Germany

Hugh Piggins

University of Manchester, United Kingdom

Philip Poronnik

University of Sydney, Australia

Mark Rasenick

University of Chicago, USA

Karen M Ridge

Northwestern University, USA

Timo Rieg

University of California San Diego, USA

Jayashree Sarathy

Benedictine University, USA

Sushil K Sarna

University of Texas, USA

Lisa M Satlin

Icahn School of Medicine at Mount Sinai, USA

Ann Schreihofer

University of North Texas Health Science Center, USA

Ole M Sejersted

University of Oslo, Norway

Kumar Sharma

University of California, USA

Katsuei Shibuki

Niigata University, Japan

Alexander V Sirotkin

Animal Research Centre Nitra, Slovakia

Sean D Stocker

Penn State College of Medicine, USA

Jun Sun

Rush University, USA

Kelly J Suter

University of Texas San Antonio, USA

Yuichiro Suzuki

Georgetown University, USA

Joel Tabak

Florida State University, USA

Michael Taggart

Newcastle University, United Kingdom

Alexei Tepikin

University of Liverpool, United Kingdom

Carole Torsney

University of Edinburgh, United Kingdom

Jolanda van der Velden

VU University, Netherlands

V K Viswanathan

University of Arizona, USA

Sandhra S Visweswariah

Indian Institute of Science, India

Jeremy PT Ward

King's College London, United Kingdom

Yoichi Ueta

University of Occupational and Environmental Health, Japan

Christopher M Waters

University Tennessee Health Science Center, USA

Moira Whyte

Royal Hallamshire Hospital, United Kingdom

John A Williams

University of Michigan Medical School, USA

Margaret Wong-Riley

Medical College of Wisconsin, USA

Charles E Wood

University of Florida, USA

Howard J Worman

Columbia University, USA

